# ZleepAnlystNet: a novel deep learning model for automatic sleep stage scoring based on single-channel raw EEG data using separating training

**DOI:** 10.1038/s41598-024-60796-y

**Published:** 2024-04-29

**Authors:** Nantawachara Jirakittayakorn, Yodchanan Wongsawat, Somsak Mitrirattanakul

**Affiliations:** 1 Institute for Innovative Learning, Mahidol University, Nakhon Pathom, Thailand; 2 Faculty of Dentistry, Mahidol University, Bangkok, Thailand; 3 Department of Biomedical Engineering, Faculty of Engineering, Mahidol University, Nakhon Pathom, Thailand; 4 Department of Masticatory Science, Faculty of Dentistry, Mahidol University, Bangkok, Thailand

**Keywords:** Computational science, Machine learning, Sleep

## Abstract

Numerous models for sleep stage scoring utilizing single-channel raw EEG signal have typically employed CNN and BiLSTM architectures. While these models, incorporating temporal information for sequence classification, demonstrate superior overall performance, they often exhibit low per-class performance for N1-stage, necessitating an adjustment of loss function. However, the efficacy of such adjustment is constrained by the training process. In this study, a pioneering training approach called separating training is introduced, alongside a novel model, to enhance performance. The developed model comprises 15 CNN models with varying loss function weights for feature extraction and 1 BiLSTM for sequence classification. Due to its architecture, this model cannot be trained using an end-to-end approach, necessitating separate training for each component using the Sleep-EDF dataset. Achieving an overall accuracy of 87.02%, MF1 of 82.09%, Kappa of 0.8221, and per-class F1-socres (W 90.34%, N1 54.23%, N2 89.53%, N3 88.96%, and REM 87.40%), our model demonstrates promising performance. Comparison with sleep technicians reveals a Kappa of 0.7015, indicating alignment with reference sleep stags. Additionally, cross-dataset validation and adaptation through training with the SHHS dataset yield an overall accuracy of 84.40%, MF1 of 74.96% and Kappa of 0.7785 when tested with the Sleep-EDF-13 dataset. These findings underscore the generalization potential in model architecture design facilitated by our novel training approach.

## Introduction

Sleep is a physiological state characterized by reduced motility and sensory responses to external stimuli across various species^1^. The quality of sleep significantly impacts both physical and mental health^2^, cognitive function^3^, and work efficiency^4^. Sleep disorders, deprivation, and disruptions from lifestyle factors such as shift work and jet-lag can lead to serious health risks, e.g., cardiovascular, respiratory, neurological, etc.^5^, and cause tremendous life-threatening incidents like vehicle and industrial accidents^6^. An estimation of 50–70 million people in the U.S. have confronted sleep problems^7^, affecting over 60% of adults^8^. Polysomnography (PSG) is the diagnostic gold standard in capturing electrical signals including electroencephalogram (EEG), electro-occulogram (EOG), electromyogram (EMG), and electrocardiogram (ECG), along with mechanical signals like airflow, sound, pressure, and body position. These signals help identify specific sleep issues, such as obstructive sleep apnea identified through respiratory events^9^, whereas snoring is observed by the sound recording signal. The sleep stage scoring process is an essential component of several assessments related to sleep quality and problems.

Sleep stage scoring, also termed sleep stage classification, is a process of labeling 30-s PSG epochs comprising EEG, EOG, and chin EMG signals. In 1968, Rechtschaffen and Kales introduced a manual for sleep stage scoring which has been called the R&K classification^10^. Later, in 2007, the American Academy of Sleep Medicine (AASM) introduced the AASM classification and is still extensively used^11^. Although with similar EEG frequency characteristics, these standards differ. According to the AASM, sleep stages include rapid eye movement (REM) and non-rapid eye movement (NREM)^11^. NREM is subdivided into N1, N2, and N3. Nightly recorded PSG is analyzed by a sleep technician, where each epoch must be manually labeled with one sleep stage and other related events which is time consuming and tremendous labor intensive. The meticulous scoring process limits patient throughput and increases workloads of sleep technicians for score finalizing. Moreover, several studies have reported varying levels of agreement among different sleep technicians^12–20^. Overall agreement levels have ranged from 61.1 to 92.2%, which indicates human errors within the scoring process. Consequently, several efforts have been made to develop automatic sleep stage scoring systems to assist technicians, hence, reducing workload, scoring duration, and human errors while accommodating more patients on sleep monitoring^21–42^.

Automated sleep stage classification systems can be categorized into two main types of learning: shallow learning, and deep learning^43^. Shallow learning involves learning a process where features extraction is based on human expert knowledge. In contrast, deep learning conducts feature extraction directly from raw data. Both approaches are then followed by classifiers to label the sleep stage.

In shallow learning, meaningful features are extracted by humans drawing upon their knowledge of both signal processing and sleep stage scoring manual. The extracted features span in time, frequency, and time–frequency domains, including non-linear features^21–27,29,35,39,41^. Various classification models are utilized to classify these extracted features ranging from traditional machine learning models such as k-nearest neighbor (kNN)^35^, support vector machines (SVM)^21,27,33^, decision tree^25^, and random forest^24,29^, to artificial neural networks (ANNs), and deep learning models which excel in the feature-based approach^23^.

A multitude of combinations between the extracted features and classification models can be generated. However, a comparative review on sleep stage classification methods^22^ highlighted five models that used traditional machine learning models as state-of-the-art models^44–48^ and conducted experiments by implementing these models on healthy subjects and REM behavioral disorder (RBD) patients. The authors pointed out that the combination of time–frequency domain and entropy features with random forest classifier obtained the highest average overall accuracy in both healthy subjects (87.06%) and RBD patients (69.05%). Another review^49^ underscored an important capability of ANNs, which are suitable for solving of non-linear problems and do not require special transformation of data prior to use. The authors reviewed that ANNs have the potential to differentiate sleep stages. One ANN system^50^ which used central EEG, two EOG, and one chin EMG channels for classifying sleep stages attained 80% overall agreement and 0.72 Cohen’s kappa coefficient in distinguishing movement time (MT), W, N1, N2, N3, and REM. However, it comes up with unexplainable in design because there are no standard guidelines to determine the number of hidden layers and neurons in the network. Most developers have found them by trial-and-error method and based on their experience^51^. Even if deep learning workflow is implemented to distinguish sleep stages based on feature-based approach^23^. The multi-layer perceptron followed by the recurrent neural network was inputted by several features in frequency domain from a single-channel EEG could obtain overall accuracy of 85.92% with macro F1-score of 80.50%.

Although sleep stage classification systems based on shallow learning have achieved superior performance, they also require extensive knowledge of signal processing and sleep stage manuals. Deep learning-based systems, on the other hand, are believed to take benefit from minimal knowledge requirements regarding signal processing and sleep stage manuals^43^. These systems can learn features directly from raw input data without prior knowledge, but the non-interpretability of the results and the more computational duration are required as trade-off. The raw data is fed into a network of filters and pooling layers to extract representative features while reducing dimensionality. Subsequently, these representative features are passed into the last layer, typically a softmax layer, to calculate the probability of each class and identify sleep stage. Deep learning models are based on artificial neural networks but differ primarily in architecture, which refers to the arrangement of neuronal connections. Various architectures including autoencoders^39,52^, deep neural networks (DNNs)^23^, convolutional neural networks (CNNs)^26,31,34,40,41,53–57^, recurrent neural networks (RNNs)^58^, and combinations among them^30,36–38,59–63^ are employed in sleep stage classification. Despite the popularity of deep learning models and their comparable performance to traditional machine learning models, accurately classifying the N1-stage remains challenging due to the similarity of N1 and REM stages, especially with single channel EEG data.

To address the similarity between N1 and REM stages, a contextual input uses surrounding epoch data for classification, termed as a many-to-one classification scheme has been introduced^31,40^. Some researchers have incorporated recurrent neural networks (RNN), particularly long short-term memory (LSTM), to capture information from neighboring temporal sequences in the classification task^27,30,32,36–38^. Evidence has showed improvement in the classification performance^39^. Bidirectional LSTM (BiLSTM) is predominantly applied to learn similar sleep stage transition, which mirrors sleep technicians who follow the AASM standard^11^. Dong^23^ combined LSTM with hand-crafted engineered features resulting in promising performance with overall accuracy 85.92%, MF1 80.50%, and F1-score for N1 was 56.31%, REM was 86.12%; however, this method used extracted features as the input. Consequently, Supratak et al.^37^ introduced DeepSleepNet for automatic sleep stage scoring based on raw single-channel EEG with automatic feature extraction. It employed a two-step training model where initially learned feature extraction of EEG signal was subsequently finetuned with BiLSTM. The results demonstrated overall accuracy of 82.00%, and MF1 of 76.90% using single-channel raw EEG signal. The F1-score for N1 and REM were 46.60%, 82.40%, respectively. Other groups utilized multiple signals, combining EEG with either EOG or EMG or both, and achieved higher F1-score of N1 and REM. Even with the addition of only one more EOG signal^52,54,64^, the highest F1-score of N1 and REM were 56% and 93%, respectively as demonstrated by Perslev et al.^64^, by incorporating just one EOG signal as the input.

According to DeepSleepNet by Supratak^37^, it was trained with two-step approach. Firstly, the representative feature extraction part was trained, followed by fine-tuning the sequential classification part. This method may incorporate the effects of sleep sequence on the feature extraction, which has already been well trained by the training data. Overfitting may occur within feature extraction due to the number of sequences in classification since sleep stage transition events are lesser than continuum events. Additionally, DeepSleepNet-Lite by Fiorillo et al.^65^ applied only the first representative feature extraction part of DeepSleepNet. It showed slightly superior performance with enormous model complexity by using 3 consecutive epochs to label the central epoch, which differs from DeepSleepNet that requires the entire night’s data. This classification can be categorized as many-to-one classification scheme^31^, and both previous and next epochs seems to contain some important features for classifying the central epoch as well. With these two ideas—the effects of sleep sequence on the feature extraction and the importance of features from the previous and the next epochs on the current epoch—it has been determined that separating training along with data manipulation can enhance the performance of sleep stage classification.

The issue of distinguishing between N1 and REM is also an important concern in this study. Typically, N1 epochs significantly comprise fewer instances compared to the others resulting in data imbalance. This imbalance can cause certain classes to be underrepresented during model training leading to reduced importance assigned to their associated features. It is sought to be addressed by adjusting the weights of specific classes to increase their importance. In other words, the importance of N1 is elevated to be equal to the sum of the others. However, this approach may have drawbacks, as it tends to prioritize the emphasized class while neglecting others, hence, introducing errors in another form. To mitigate this, it is necessary to employ multiple models, which each model focuses on an individual class to ensure equal importance across all classes. This model design cannot be trained using either end-to-end or fine-tuning processes. Therefore, separating training is introduced and employed to enable the training of designed model and evaluate its performance.

As suggested in the literature review^43^, evaluating a model’s performance by solely overall accuracy is insufficient, at least agreement with the scorer is required. The interrater reliability among sleep technicians has been quantified using Cohen’s kappa coefficient in meta-analysis study which resulted in an overall value of 0.76^66^. Additionally, Cohen’s kappa coefficients for each stage vary with values reported as 0.70, 0.24, 0.57, 0.57, and 0.69, for W, N1, N2, N3, and REM respectively. These findings serve as rough criteria for assessing consistency between automatic sleep stage classification and human scorers. Previous studies have developed models based on consensus scoring among five sleep technicians on DOD-H dataset^19,20^. Fiorillo et al.^19^ reported a Cohen’s kappa coefficient of 0.82 among five sleep technicians while Guillot et al.^20^ demonstrated a 79.9% agreement. These studies have estimated the consensus-scoring through soft agreement using their respective methods. In this study, the interrater reliability (consistency) between the developed model and sleep technicians through statistical analysis is also focused on.

The primary objective of this study is to develop a novel deep learning model for automated sleep stage scoring utilizing single-channel raw EEG data and separating training to enhance model performance and efficiency. This study also aims to investigate the interrater reliability of sleep stage scoring across the dataset, developed model, and other sleep technicians. This comparison may be used to evaluate the consistency and agreement among raters in determining sleep stages while obtaining clinical applicability of the developed model. Eventually, cross dataset validation will be used to validate the developed model’s generalization.

## Results

**Figure 1:**
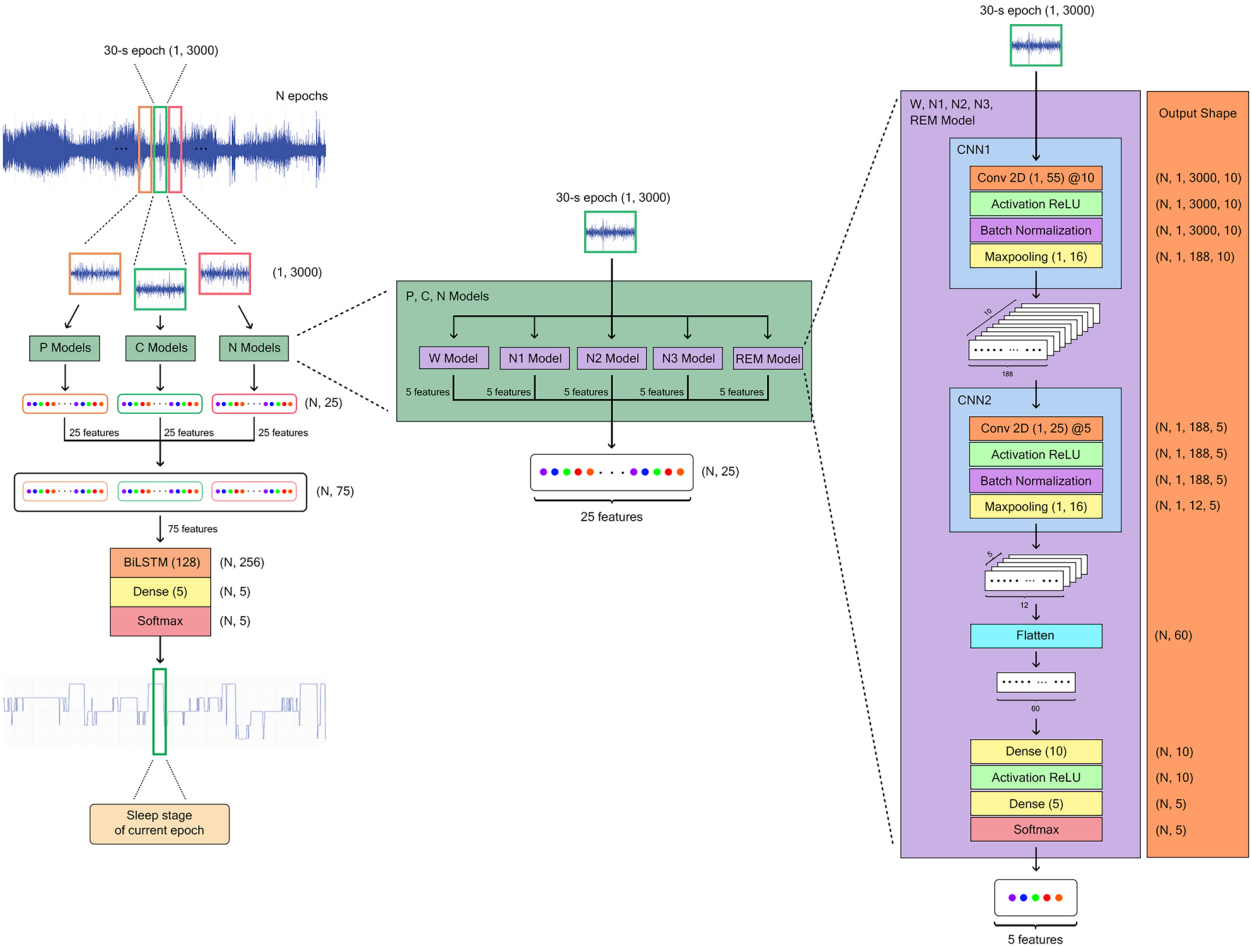
Overview of ZleepAnlystNet. The left column illustrates the prediction process from a complete EEG signal sequence to an entire sleep stage sequence. Three sets of CNN models called P-, C-, and N-models are exhibited. Color frames representing signal shifting for each set of CNN models. The middle column displays the composition of class-specific models in each set of CNN models. The right column presents the architecture of each CNN model in class-specific models. While all class-specific models share the same architecture, they vary in the weights of loss function.

**Figure 2:**
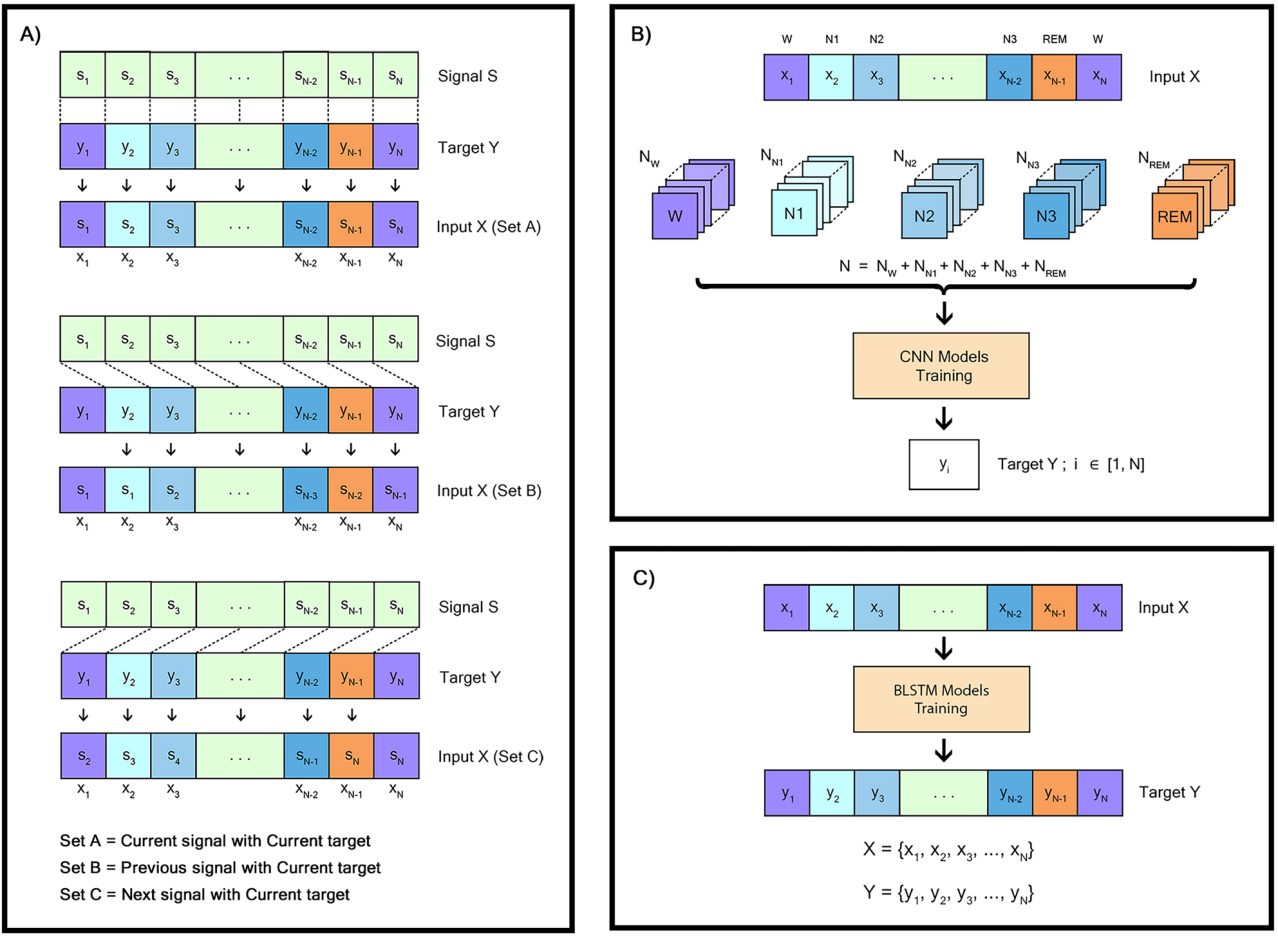
Data manipulation and training processes. (**A**) Data manipulation process by involving the combination of signal S and target Y, resulting in input X. (**B**) Training of CNN separated by sleep stage. The input X of CNN models are one whole sequence of EEG signal. (**C**) Training of BiLSTM model. The input X for the BiLSTM model is the output of the trained CNN models arranged in the original sequence of EEG signal, generated from feeding the entire EEG signals. Signal manipulation and specific set of the models, P-, C-, and N-models are utilized.

The model named ZleepAnlystNet consisted of two main parts: representative feature extraction and sequential sleep stage classification (Fig. 1 on the left column), detailed in the ‘Methods’ section. These two parts underwent separate training during the model training procedure (Fig. 2B, C). The developed model was trained and evaluated using Sleep-EDF-13 and Sleep-EDF-18 datasets. Model evaluation involved subject-wise k-fold cross-validation with k values of 20 and 10, respectively.

### Developed model performance

**Figure 3:**
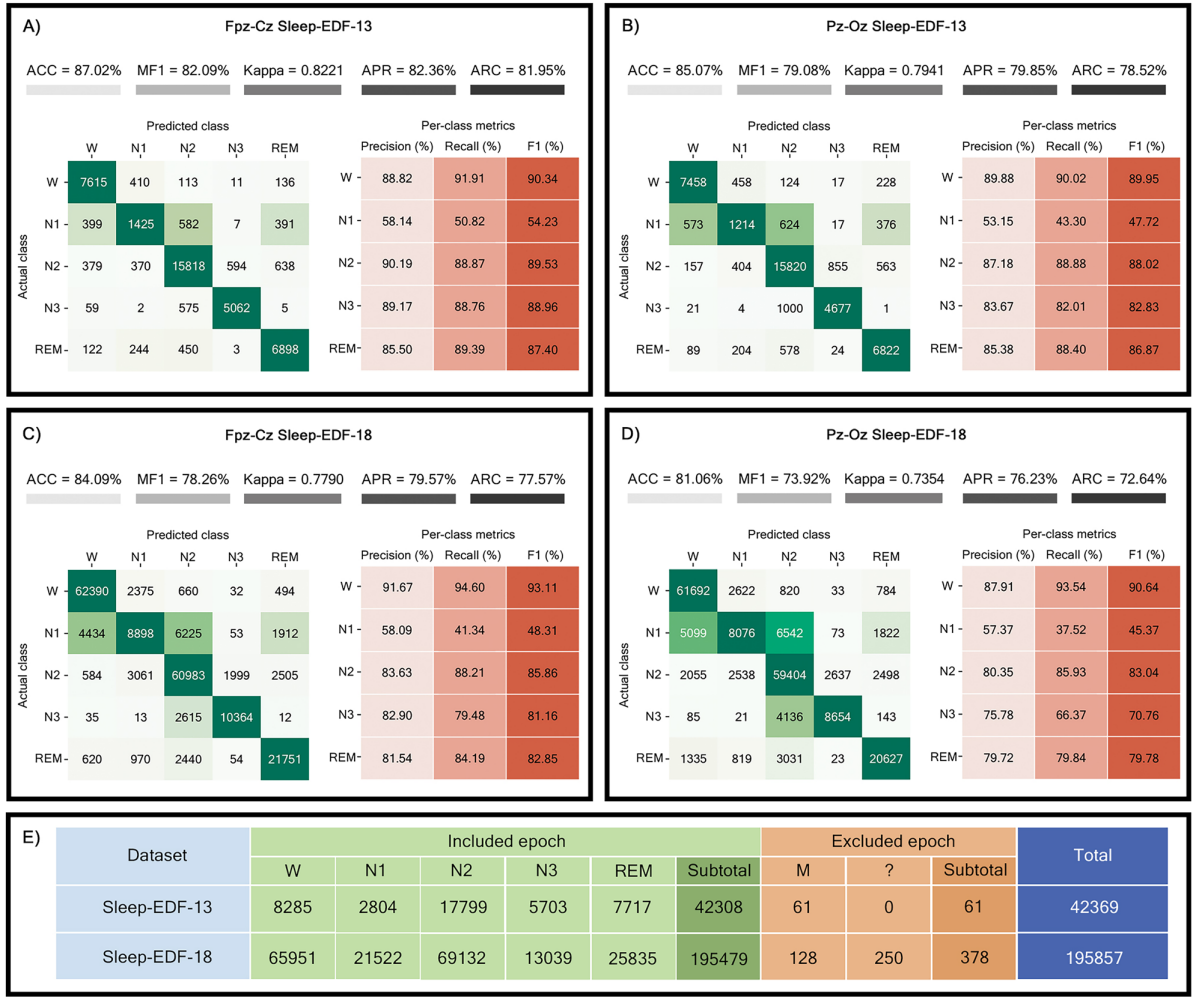
Model performance metrics. Confusion matrices, overall evaluation metrics, and per-class evaluation metrics of the developed model trained by the Sleep-EDF dataset. (**A**) Fpz-Cz channel of Sleep-EDF-13. (**B**) Pz-Oz channel of Sleep-EDF-13. (**C**) Fpz-Cz channel of Sleep-EDF-18. (**D**) Pz-Oz channel of Sleep-EDF-18. (**E**) Dataset details and numbers of epochs utilized in model training and evaluation.

Figure 3A–D depicts the performance of the model under various input conditions. The Fpz-Cz channel of the Sleep-EDF-13 dataset exhibited the highest overall accuracy (87.02%), MF1 (82.09%), average precision (82.36%), and average recall (81.95%). Across all input conditions, the N1-stage consistently showed the lowest per-class F1. The model tended to misclassify the N1-stage as W, N2, and REM across all input conditions. However, when compared to other conditions, the per-class F1-score of the Fpz-Cz channel of the Sleep-EDF-13 dataset was the highest, reaching 90.34%, 54.23%, 89.53%, 88.96%, and 87.40% for W, N1, N2, N3, and REM stages, respectively. Figure 3E provides an overview of the total number of epochs used, including both included and excluded epochs for model evaluation.

### Comparison between performances of developed model and existing models

For a systematic comparison, it is crucial to control the environmental conditions, which includes the dataset used, EEG input specifications such as channel and total number of epochs, and evaluation strategies. Hence, the Slee-EDF-13 dataset was selected due to its limited records and consistent subject amount facilitating leave-one-subject-out cross-validation for equitable comparisons across studies. Models with single-channel data input were exclusively considered to ensure fair performance comparisons with our proposed model.

From Table 1, it became evident that the developed model, ZleepAnlystNet, achieved the highest overall accuracy and MF1 percentage in both conditions, utilizing Fpz-Cz and Pz-Oz inputs from the Sleep-EDF-13 dataset. Specifically, ZleepAnlystNet attained an overall accuracy of 87.02% and 85.07%, and MF1 of 82.09% and 79.08%, respectively for Fpz-Cz and Pz-Oz inputs. The kappa coefficient indicating superior performance equaled 0.8221, which equivalent to EEGSNet employing spectrogram image input^28^. Notably, the per-class F1-score demonstrated ZleepAnlystNet’ superior performance in classifying sleep stages N1, N2, and N3 using Fpz-Cz input with values of 54.23%, 89.53%, and 88.96%, respectively. Furthermore, ZleepAnlystNet achieved F1-score of 90.34% and 87.40% for W and REM stages, respectively, using Fpz-Cz input. However, for the Pz-Oz input, ZleepAnlystNet exhibited the highest performance in REM-stage compared to existing models utilizing the same input.

**Table 1:** Comparisons of model performance including numbers of training parameters, overall evaluation metrics, and per-class evaluation metrics between the developed model and the existing models in the literature.

Model	References	Training parameters	Architecture	Input channel	Input type	ACC	MF1	Kappa (κ)	F1 score (%)				
						(%)	(%)		W	N1	N2	N3	REM
**ZleepAnlystNet**	**–**	**2,48,500**	CNN + BiLSTM	Fpz-Cz	Time series	**87.02**	**82.09**	**0.8221**	90.34	**54.23**	**89.53**	**88.96**	87.40
DeepSleepNet	^37^	~ 24.7 M	CNN + BiLSTM	Fpz-Cz	Time series	82.00	76.90	0.76	84.70	46.60	85.90	84.80	82.40
XSleepNet	^63^	~ 5.8 M	BiLSTM	Fpz-Cz	Time series	86.30	80.60	0.81	90.20	51.80	88.00	86.80	83.90
SleepEEGNet	^30^	~ 2.6 M	CNN + BiLSTM	Fpz-Cz	Time series	84.26	79.66	0.79	89.19	52.19	86.77	85.13	85.02
SingleChannelNet	^69^	n/a	CNN	Fpz-Cz	Many-to-Many	86.20	n/a	0.81	n/a	n/a	n/a	n/a	n/a
Joint Classification	^31^	n/a	CNN	Fpz-Cz	One-to-Many	81.90	73.80	0.74	n/a	n/a	n/a	n/a	n/a
TinySleepNet	^38^	~ 1.3 M	CNN + LSTM	Fpz-Cz	Time series	85.40	80.50	0.80	90.10	51.40	88.50	88.30	84.30
EEGSNet	^28^	~ 0.6 M	CNN + BiLSTM	Fpz-Cz	Spectrogram Image	86.82	81.57	**0.82**	**90.76**	52.41	88.78	87.00	**87.89**
DeepSleepNet-Lite	^65^	~ 0.6 M	CNN	Fpz-Cz	Many-to-One	84.00	78.00	0.78	87.10	44.40	87.90	88.20	82.40
IITNet	^36^	n/a	CNN + BiLSTM	Fpz-Cz	Many-to-One	83.90	77.60	0.78	87.70	43.40	87.70	86.70	82.50
Yang B., et al	^57^	n/a	CNN + CRF	Fpz-Cz	Time series	85.20	77.70	0.79	88.90	40.60	87.70	86.70	85.00
Yang B., et al	^57^	n/a	CNN + BiLSTM + CRF	Fpz-Cz	Time series	85.20	78.10	0.79	88.10	42.40	87.20	87.80	85.00
**ZleepAnlystNet**	**–**	**2,48,500**	CNN + BiLSTM	Pz-Oz	Time series	**85.07**	**79.08**	**0.79**	89.95	**47.72**	**88.02**	**82.83**	**86.87**
SleepEEGNet	^30^	~ 2.6 M	CNN + BiLSTM	Pz-Oz	Time series	82.83	77.02	0.77	**90.27**	44.64	85.74	81.55	82.88
DeepSleepNet	^37^	~ 24.7 M	CNN + BiLSTM	Pz-Oz	Time series	79.80	73.10	0.72	88.10	37.00	82.70	77.30	80.30

The input channel was separated as either Fpz-Cz or Pz-Oz. Bold values indicate the highest value for each metric.

### Efficacies of model training approach and data manipulation

To investigate the efficacies of training approach and data manipulation, two scenarios were simulated: scenario A—separating training and end-to-end training, and scenario B—several combinations of data manipulation sets. In both scenarios, the Fpz-Cz channel of the Sleep-EDF-13 dataset was utilized as input.

For scenario A, the 15 individual CNN models were changed into a single CNN model for feature extraction without employing a specific weighted loss function tailored to sleep stage. Additionally, a single BiLSTM model received input from 5 features per epoch extracted by the CNN model. The architectural design of the CNN model was illustrated in Fig. 1 (on the right column). In the case of separating training approach, the single CNN model underwent training with data manipulation set A. Following this, the BiLSTM model was trained with the output from the CNN model. Conversely, in the case of end-to-end training, the same CNN and BiLSTM models were interconnected into CNN + BiLSTM. The CNN + BiLSTM model was trained using data manipulation set A. Further details were fully provided in the ‘Methods’ section. As summarized in Table 2, separating training yielded notably superior performance (exceeding 5%) across both overall and per-class metrics.

**Table 2:** Performance of sleep stage classification using various CNN training approaches.

Scenario detail	Overall metrics					Per-class metrics														
	ACC	APR	ARC	MF1	Kappa	Precision					Recall					F1				
						W	N1	N2	N3	REM	W	N1	N2	N3	REM	W	N1	N2	N3	REM
Scenario A: Efficacy of separating training and end-to-end training																				
· Separating training	86.27	81.19	80.51	80.78	0.8113	87.13	53.97	89.23	89.06	86.55	90.73	46.54	88.95	87.67	88.66	88.90	49.98	89.09	88.36	87.59
· End-to-end training	79.84	75.26	73.38	74.02	0.7225	85.06	49.73	84.17	87.22	70.10	82.46	39.51	84.22	77.28	83.44	83.74	44.04	84.20	81.94	76.19
Scenario B: Efficacy of sets of data manipulation in CNN training																				
· The Current models (set A) only	86.76	82.03	81.67	81.78	0.8184	88.14	57.48	89.85	88.76	85.90	92.85	49.86	88.50	89.74	87.38	90.44	53.40	89.25	86.63	86.76
· The Previous models (set B) only	86.44	81.93	81.27	81.55	0.8136	91.19	57.13	88.70	88.65	83.99	91.32	50.46	88.74	88.06	87.75	91.26	53.59	88.72	88.35	85.83
· The Next models (set C) only	86.70	81.95	81.10	81.39	0.8173	90.94	57.07	89.42	89.13	83.21	92.11	46.79	88.57	87.71	90.32	91.52	51.52	88.99	88.41	86.62
· The Current models and the Previous models (set A and set B)	86.85	82.03	82.00	81.99	0.8198	88.99	56.50	90.04	88.51	86.12	91.93	51.75	88.54	89.43	88.36	90.44	54.02	89.28	88.97	87.23
· The Current models and the Next models (set A and set C)	86.69	82.08	81.46	81.67	0.8174	87.88	58.21	89.54	88.51	86.24	92.30	49.04	88.56	89.15	88.23	90.03	53.23	89.05	88.83	87.23
· The Previous models and the Next models (set B and set C)	86.67	82.22	81.28	81.65	0.8168	90.31	58.57	89.00	88.82	84.38	91.48	48.86	88.94	88.32	88.78	90.89	53.28	88.97	88.57	86.52
· The developed condition (set A, set B, and set C)	87.02	82.36	81.95	82.09	0.8221	88.82	58.14	90.19	89.17	85.50	91.91	50.82	88.87	88.76	89.39	90.34	54.23	89.53	88.96	87.40

Scenario A explored different training approaches. Scenario B examined combinations of data manipulation.

For scenario B, data manipulation sets were investigated. Our proposed model consisted of three sets of CNN models: P-models, C-models, and N-models. Each set comprised 5 class-specific CNN models, all sharing the same architecture (Fig. 1 on the right column) but varying in the loss functions used during model training. The distinguishing factor among the three sets of the CNN models was the input they received. The C-models, referred to as the set of the ‘Current’ models, were trained using data manipulation set A (Fig. 2A), while the P- and N-models utilized sets B and C, respectively. Within this scenario, different combinations of data manipulation were investigated. Each combination involved training the BiLSTM model with sets of CNN models corresponding to the manipulated input data. For example, in the case of using only set A, the C-models were trained with data manipulation set A, and the BiLSTM model was subsequently trained using the output of the trained C-models generated by feeding data manipulation set A. Only 5 features per epoch, not all datapoints of the signal, were sent through the BiLSTM model. On the other hand, in the case of combining set A and set B, the C-models were trained with data manipulation set A while the P-models were trained with set B. During the BiLSTM training, 10 features per epoch were sent through the model. The specific hyperparameters were explained in the ‘Methods’ section. In total, seven combinations were evaluated including: only set A; only set B; only set C; set A + set B; set A + set C; set B + set C; and set A + set B + set C. Table 2 shows that overall metrics displayed slightly different performance (within 1% of difference) upon each condition, while the developed condition exhibited the highest accuracy of 87.02% with MF1 of 82.09%. In terms of per-class metrics, N1 classification consistently exhibited the lowest performance across all conditions. The highest per-class F1-score was found in the developed condition, while the second highest was found in sets A and B condition which was a combination of two sets of the ‘Current’ and the ‘Previous’ models.

### The interrater reliability

**Figure 4:**
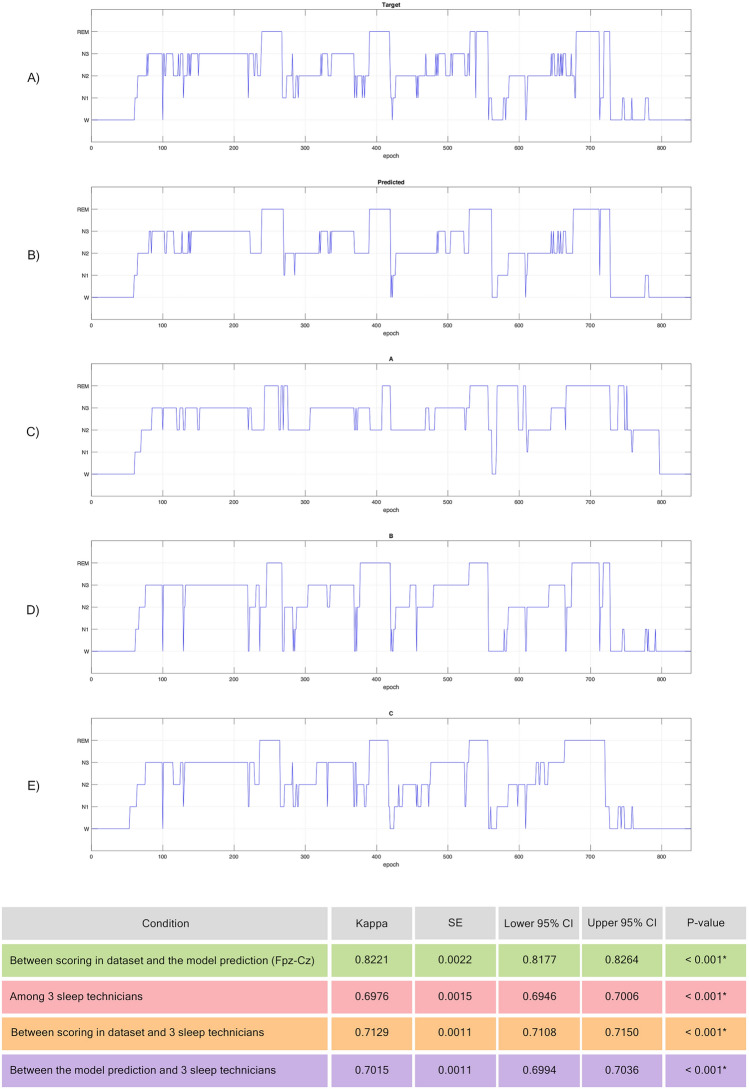
Comparison of sleep stage scoring among dataset, model predictions, and three other sleep technicians. (**A**) Dataset scoring. (**B**) Model predictions. (**C**)–(**E**) sleep technicians scoring. * indicates significant differences.

Figure 4A–E illustrates the sleep architecture of the same recording scored by different scorers: A) depicts the sleep stage as scored in the dataset, B) shows the model prediction, and C) to E) represent the sleep stage scored by three other sleep technicians. In the first condition, consistency agreement between the dataset scoring and the model prediction was strong (κ = 0.8221, 95% CI of κ = 0.8177 − 0.8264, *p*-value < 0.001). In the second condition, interrater agreement among three sleep technicians was moderate (κ = 0.6976, 95% CI of κ = 0.6946–0.7006, *p*-value < 0.001). In the subsequent condition, the interrater agreement between scoring in the dataset and 3 sleep technicians was also moderate (κ = 0.7129, 95% CI of κ = 0.7108–0.7150, *p*-value < 0.001). Lastly, the consistency agreement between the model prediction and the three sleep technicians was moderate (κ = 0.7015, 95% CI of κ = 0.6994–0.7036, *p*-value < 0.001).

### Cross-dataset validation

Cross-dataset validation was implemented using 200 recordings sampled from SHHS1 and SHHS2, 100 records from each dataset, ensuring comparability between results and the developed conditions. As mentioned, the primary objective of this study was to focus on separating training approach so the systematic evaluation across dataset was not provided. However, 200 records were sufficient for a preliminary investigation of the idea. After inputting 200 records into the models in all folds, the best fold in each condition was further tested using the full SHHS1 dataset, consisting of 6,441 records, as pilot study. The number of SHHS dataset epochs used in this study, in the case of 200 records, is given in Supplementary Fig. S1C.

Supplementary Tables S1–S8 present the evaluation results of 60 models trained using both Fpz-Cz and Pz-Oz of the Sleep-EDF-13 and the Sleep-EDF-18 datasets on both C3-A2 and C4-A2 of the SHHS dataset. Totally, 8 cases of the combinations between datasets used for model training and evaluation were generated which comprised of training from 2 channels of both Sleep-EDF-13 and Sleep-EDF-18 datasets and evaluated on 2 channels of SHHS dataset. The two highest overall and per-class model evaluation metrics were observed in combination case 4 (Supplementary Table S4) and case 5 (Supplementary Table S5). According to case 4, 20 models were trained with the Pz-Oz channel of the Sleep-EDF-13 dataset and evaluated with the C4-A1 channel of the SHHS dataset, 200 records. The overall metrics for model evaluation were as follows: 64.25% for overall accuracy (ACC); 56.40% for MF1; 0.4965 for Kappa; 62.38% for overall average of precision (APR); and 57.14% for the overall average of recall (ARC). For per-class metrics of model evaluation, the following per-class F1-score was reported due to sleep stage: W (61.75%); N1 (20.24%); N2 (69.03%); N3 (64.60%); and REM (66.36%). According to case 5, 10 models were trained with the Fpz-Cz channel of the Sleep-EDF-18 dataset and evaluated with the C3-A2 channel of the SHHS dataset, 200 records. It revealed the following overall metrics: 63.06% for ACC; 53.44% for MF1; 0.4859 for Kappa; 63.22% for APR; and 57.12% for ARC. The per-class F1-score was as follows: W (62.17%); N1 (26.04%); N2 (71.75%); N3 (42.86%); and REM (64.37%). The average values of other overall and per-class metrics for each case with their standard deviations are exhibited in Supplementary Figs. S2 and S3. When running with full SHHS1 dataset, the best model in 20 models from case 4, fold-13, demonstrated an overall accuracy of 67.51%, MF1 of 59.43%, Kappa of 0.6148, APR of 64.91%, and ARC of 58.19%. The per-class F1-score was 67.00%, 25.02%, 72.10%, 67.14%, and 65.89%, for W, N1, N2, N3, and REM respectively (Supplementary Table S4, the bottommost). For case 5, fold-9, the model showed an overall accuracy of 67.45%, MF1 of 57.35%, Kappa of 0.6100, APR of 63.35%, and ARC of 58.94%. The per-class F1-socres were 70.08%, 29.59%, 74.55%, 45.80%, and 66.76%, for W, N1, N2, N3, and REM respectively.

Consequently, the developed model was newly trained with 200-recordings SHHS dataset to evaluate its architecture potentiality. Both the C3-A2 and the C4-A1 channels were separately trained and model performance was then assessed.

Supplementary Fig. S1A, B represent the model performance of two input conditions, the C3-A2 and the C4-A1 channels of the SHHS dataset. The lowest overall and per-class metrics were observed in the C4-A1 channel, with ACC of 83.75%, MF1 of 73.40%, Kappa of 0.7682, APR of 77.12%, and ARC of 71.56%. Per-class F1-score due to each class was as follows: W (89.42%), N1 (33.02%), N2 (84.23%), N3 (76.76%), and REM (83.56%). The N1-stage exhibited the lowest per-class F1-score, while the N2-stage showed the highest.

## Discussion

This study primarily aims to develop a novel sleep stage scoring model based on single-channel raw EEG data using a novel training approach called separating training. Model performance was assessed using the Sleep-EDF-13 and the Sleep-EDF-18 datasets incorporating the Fpz-Cz and the Pz-Oz channels input separately. As secondary objective, interrater reliabilities were compared among model prediction, dataset scoring, and scoring by three other well-trained sleep technicians. Additionally, to investigate the feasibility of generalization, cross-dataset validation was conducted using the SHHS dataset comprising the C3-A2 and the C4-A1 channels. The model’s potential was evaluated by training with the SHHS dataset, using the C3-A2 and the C4-A1 channels independently.

The developed model, ZleepAnlystNet, comprises two main parts: the feature extraction part and the sleep stage classification part. For feature extraction, 15 CNN models were utilized, organized into 3 data manipulation sets and focused on capturing time-invariant features. These sets, named set A, set B, and Set C, integrated raw EEG signals from different epochs with dataset scoring. In set A, EEG signal from the current epoch was mapped with the sleep stage of the current epoch while in set B and C, EEG signal from the previous and the next epochs were also mapped with the sleep stage of the current epoch. Each data manipulation set was used to train their corresponding set of CNN models: C-models, P-models, and N-models using set A, set B, and set C, respectively. In C-, P-, and N-models comprised five class-specific models for each sleep stage: W, N1, N2, N3, and REM models. During model training, distinct weights of loss function corresponding to each sleep stage were applied, which resulted in the separate training of 15 individual CNN models. Sleep stage classification involved utilizing a single BiLSTM with 128 hidden units that was informed by the output of the trained three sets of CNN models. To evaluate the developed model, k-fold cross-validation was employed using both datasets; 20-fold for Sleep-EDF-13 and tenfold for Sleep-EDF-18.

The justification for formulating the suggested model, which incorporates the distinct separating training technique, stems from a range of systematic considerations. Initially, an inspiration was drawn from Phan et al.^31^ who is one of the pioneers, as far as our knowledge, in organization classification schemes by dividing them into one-to-one, many-to-one, many-to-many, and they suggested one-to-many classification scheme in their work, Joint Classification. This model reached an overall accuracy of 81.9% and MF1 of 73.8%. The concept of many-to-one classification in sleep stage scoring has been adopted since it mimics the recommendation following the guidelines^11^. Subsequently, the model called DeepSleepNet-Lite proposed by Fiorillo et al.^65^, which employed the first representative feature extraction part of DeepSleepNet^37^, employed many-to-one classification scheme using only three consecutive PSG-epoch and achieved high performance in the Sleep-EDF-13 dataset with an overall accuracy of 84.0% and MF1 of 78.0%. These two models, Joint Classification and DeepSleepNet-Lite, extracted the representative features then classified the extracted features into sleep stage sequence, and achieved high performance. Therefore, it seems that previous and next epochs also have some important or key features to classify sleep stage of the current epoch. Moreover, DeepSleepNet proposed by Supratak et al.^37^ and SleepEEGNet proposed by Mousavi et al.^30^ were the models that had BiLSTM connected with CNNs for extracting the representative feature following by classification of sleep stage with the involvement of temporal information. The former model obtained an overall accuracy of 82.0% and MF1 of 76.9% while the latter model reached 84.26% and 79.66%, respectively in the Sleep-EDF-13 dataset. SleepEEGNet also came up with higher per-class performance (F1) in N1-stage at 52.19% which was trained from the Fpz-Cz channel. This led to the conclusion to our rationale that the temporal information had a huge impact on the improvement of the model’s performance. Supratak et al.^37^ was the first group that introduced two-step training in model training process. This training begins with training the CNNs and then fine-tuning with BiLSTM but adjusting the learning rate of CNNs by reducing it during the fine-tuning. With this, DeepSleepNet initially learns the representative features from PSG epoch. Subsequently, the BiLSTM was trained while the weights of CNNs remain to be trained simultaneously. It can be assumed in our thought that the representative features receive more attention during the model training process. Next, SleepEEGNet by Mousavi et al.^30^ is one of the earliest groups to address the issue of data imbalance by modifying the loss function. This modification led to a significantly high F1-score of 52.19% for N1-stage. Its structure was a CNN interconnected with BiLSTM with an attention decoder. Furthermore, our consideration extends to an alternative model known as EEGSNet, which was introduced by Li et al.^28^ that preprocessed the raw data into spectrogram, time–frequency features. The model training was end-to-end with an auxiliary classifier so the CNN model for feature extraction received weight-adjustment during the model training process from two main pathways, the one from auxiliary classifier and another one from sequence classifier. The former, from auxiliary classifier, indicates that the model was trained from the current epoch while the latter indicates that the model was trained to adapt itself to sleep stage sequence. EEGSNet reached an overall accuracy of 86.82% and MF1 of 81.57% which were higher than the earlier mentioned models; moreover, per-class F1-score for N1-stage was 52.41%. However, from our point-of-view, the adjustment of CNN models’ weights based on temporal information imposes additional learning tasks on CNNs, as they must learn both epoch patterns and sequences. This drastically increases the function on CNNs required, leading to more complexity, and requiring more data for training in which overfitting potentially occurs. EEGSNet^28^ dramatically mitigates the impact of temporal information by incorporating an auxiliary classifier to aid in weight adjustment during training. Additionally, it utilizes two residual connection blocks allowing weight adjustment to propagate up to the topmost block. This enables effective training of the model even with considerable model depth. Both DeepSleeNet^37^ and SleepEEGNet^30^ share a similar CNN architecture which is two-branch hierarchical convolution blocks connected by two layers of BiLSTM. However, DeepSleepNet included shortcut connections via a fully connected layer which resulted in a higher model complexity of approximately 24.7 million parameters while SleepEEGNet consumed around 2.6 million parameters.

With all the aforementioned factors, it was considered that the incorporation of the representative features from both previous and next epoch should be included in the sleep stage classification of the current epoch; therefore, three sets of the CNN models named C-, P-, and N-models were generated in accordance with three sets of data manipulation called set A, set B, and set C, respectively for three sets of the CNN models. Furthermore, data imbalance problems among sleep stages should be addressed with the adjustment of loss function. Consequently, the weighted loss functions, which were specific to each sleep stage, were developed resulting in class-specific models. The class-specific models were composed of W, N1, N2, N3, and REM according to the sleep stage. These five class-specific models were then involved in the three sets of the CNN models upon data manipulation. The inclusion of an auxiliary multi-task classifier in the EEGSNet^28^ training regimen suggests that isolating and training each component individually could lead to increased efficiency. Coupled with the adjustment of weights for each class using weighted loss functions, necessitated the application of separating training because of both the idea and the model’s structure. While concerns regarding time consumption in real-world applications may arise due to this approach, our study did not focus on this aspect as the model was designed for offline prediction, so systematic experiments related to time consumption are not conducted. However, it is feasible to make a real-time one since the result of set A + set B indicated. Furthermore, due to the model’s structure utilizing future epochs in classification, it is non-causal system, so the real-time prediction is impossible upon the definition. However, the time spent in feed-forward process was recorded, which was 0.136 s per record for each of the 15 CNN models, totaling 2.04 s. Additionally, 0.58 s were spent on the BiLSTM model. This made the total of 2.62 s per 1 record, approximately.

According to the results, training the developed model with the Fpz-Cz channel of the Sleep-EDF-13 dataset showed slightly superior or comparable performance to the existing models. The overall metrics exhibited an overall accuracy of 87.02%, MF1 of 82.09%, and Kappa of 0.8221 while the per-class F1-score indicated 90.34%, 54.23%, 89.53%, 88.96%, and 87.40% for W, N1, N2, N3, and REM stages, respectively. The kappa coefficient of 0.8221 indicated strong reliability according to McHugh^67^ or near-perfect agreement according to Landis and Koch^68^. This result supports that the developed model demonstrated high-level performance. However, a decrease in model performance in N1-stage is anticipated due to data imbalance, which has been a major concern in automatic sleep stage scoring model development and confronted by several studies^30,31,33,37,69^. The Pz-Oz EEG channel’s performance was 3–5% lower than Fpz-Cz across metrics. This performance trend—superior overall and per-class metrics but weaker N1 results, and Pz-Oz underperforming Fpz-Cz—was consistent for both the Sleep-EDF-13 and the Sleep-EDF-18 datasets. Therefore, comparison of the developed model performance with existing models was mainly performed by using the Fpz-Cz channel of the Sleep-EDF-13 dataset as input because it offers direct comparison, in which the numerical data reported in across studies with leave-one-subject-out cross-validation strategy. Another rationale is that several existing models have reported their models’ performance with the Fpz-Cz channel of the Sleep-EDF-13 dataset for benchmarks.

Performance comparison between the developed and existing models was necessary to evaluate its effectiveness. However, all randomized factors must be controlled, for example the subject in the evaluation. For obvious comparison, the dataset condition must remain consistent across all models. The selected existing models included DeepSleepNet^37^, SleepEEGNet^30^, SignleChannelNet^69^, Joint Classification and Prediction Model^31^, and EEGSNet^28^. All mentioned models utilized the Sleep-EDF-13 dataset as input for training and evaluation. Majority of them utilized and reported merely the Fpz-Cz channel for evaluation, except SleepEEGNet^30^ and DeepSleepNet^37^ having reported both. The results revealed that the developed model showed slightly superior or comparable performance in all overall metrics including accuracy, MF1, and kappa coefficient among all existing models across both channels. For per-class metrics, stage N1, N2, and N3 exhibited the highest performance but stage W and REM showed marginally 0.40% lower than EEGSNet^28^. This suggests that the developed model may be used for automatic sleep stage scoring and could redefine a new benchmark performance for sleep stage scoring model development. As for Pz-Oz, the proposed model has the highest performance among the existing models, except for sleep stage N1 with 0.32% lower than SleepEEGNet^30^. This emphasizes that the developed model has high performance for sleep stage scoring using single-channel raw EEG data without preprocessing procedures.

The developed model's enhanced performance is attributed to two main factors: separating training approach and addressing data imbalance with specific loss function weights. By differentiating feature extraction and sequential classification, separating training may improve performance by allowing targeted training on essential representative features without the influence of sleep stage sequences. According to end-to-end training, the hyperparameters of the feature extraction model may acquire certain effects from the training of sequential sleep stage classification. This issue was avoided by separating training. Furthermore, using specific weighted loss functions to alleviate data imbalance ensured improved feature extraction. The obvious evidence of this issue can be found in direct comparison between scenario A case of separating training and scenario B case of the current models (set A) only. Since the representative features of each sleep stage are different, the weighted loss function specific to each sleep stage provided better features for each sleep stage. Together, these strategies enable our model to achieve high performance, potentially setting a new benchmark. Moreover, the separating training and model design helped mitigate overfitting by reducing model complexity. The designed CNN models extract distinct features for each epoch without learning the sleep stage sequence simultaneously, requiring fewer hyperparameters than other models. One CNN model contains 2,500 trainable parameters. There are 15 CNN models in total making 37,500 trainable parameters. Combining with 211,000 trainable parameters of one layer of BiLSTM results in a total of 248,500 trainable parameters. Whereas the other models revealed 10-times higher trained variables of 1.3 M to 24.7 M^70^. Despite this, the developed model performance is comparable to existing models.

To understand the impact of training approaches, an analysis was conducted comparing separating training to end-to-end training. Similar conditions like CNN models, dataset, EEG channel, and evaluation procedure were applied with different training approaches. The results indicated that separating training provided higher model performance than end-to-end training. Moreover, the efficacy of the loss function weights was also obviously indicated by comparison between separating training case in scenario A and the Current data manipulation set case in scenario B. The results revealed that performance in N1-stage in loss function weights case reached higher performance. Furthermore, the influence of various data manipulation sets on model training and prediction was examined. Although different data manipulation sets and their combinations were inputted into the model, its performance difference was under 1% for most evaluation metrics. This suggests that the developed model has potential for real-time scoring, especially with data manipulation sets A and B utilizing past and current EEG data, but the architecture must be changed to causal system. However, sleep stage scoring is typically conducted by sleep technicians, hence, model performance should be juxtaposed with technician performance.

Results from the three sleep technicians scoring the Sleep-EDF-13 dataset revealed a moderate agreement upon McHugh threshold^67^, deemed acceptable for medical applications. The findings emphasized human error issues that generally rose in the AI field. Further analysis comparing dataset scores with the three sleep technicians yielded consistent moderate agreement. However, the developed model showed strong agreement with the dataset, indicating that it accurately learned the scoring procedure. An evaluation of consistency reliability between the model and the three sleep technicians found comparable results. This suggests that the developed model could be a valuable tool to aid sleep technicians in sleep stage classification. In addition, several studies have developed their models based on multi-scored dataset^19,20^ by training with consensus calculated among sleep technicians. This point is of interest to include heterogeneity of sleep technicians in the model training and requires further study.

To assess the developed model's generalization in preliminary, cross-dataset validation was performed using all trained models from Sleep-EDF and SHHS datasets. The results displayed a lower performance compared to the original condition. However, when newly trained with the randomized SHHS dataset, the performance was comparable to the initial condition. This suggests the developed model exhibits generalizability but requires training on a large and diverse dataset to achieve it. Training on a small dataset appears insufficient for achieving generalization. Possible reasons include different recording equipment, acquisition criteria, and montage variations in recording process as mentioned in the literature^43^. These differences mean a single training environment might not capture all signal variations. Including both the large-size dataset and the heterogeneity of the training data enables more generalizability, evidence with Supplementary Fig. S1 and the literature^52,64^.

Our proposed model and separating training approach bring multiple distinct benefits to the task of automatic sleep stage classification. Notably, the model boasts easy scalability. It can be improved simply by assessing features to gauge their importance for each class. This enables the elimination of non-essential elements and the integration of other significant features, thereby streamlining progress on a variety of issues. Additionally, the model design enables error analysis to provide valuable insights into its performance and enables targeted improvements to enhance accuracy. Moreover, the model demonstrates a trend towards specificity in particular class. One potential method for further improvement is to selectively enhance accuracy in specific classes, particularly focusing on the N1-stage. Even post-processing during the inference phase as employed by Yang et al.^57^ can be applied to improve the performance. Despite these advantages, our proposed model does have certain limitations. The lengthy training process is a huge drawback. Training our model requires a substantial amount of time because it involves training multiple models separately; however, parallel processing is enabled for both training and implementation of the proposed model.

Finally, the study’s important limitations consist of 2 main concerns. Firstly, the interrater reliability can be conducted merely using the Sleep-EDF-13 dataset. However, the remaining datasets including Sleep-EDF-18 and 200 recordings of SHHS were absent due to the high number of recordings within the dataset. Nonetheless, the Sleep-EDF-13 dataset has 42,369 epochs which are sufficient to assess interrater reliability. Secondly, the developed model is unable to explain the extracted features. The conventional machine learning model aims to perform classification tasks using engineered-base features, thus all extracted features can be defined and explained by humans. However, the features in this study were performed by the developed model utilizing raw EEG signal. The developed model learned the features in raw time-series; therefore, it remains unexplainable as unknown features. The thorough comprehension of model behavior and its learnt features is one of the active study areas in deep learning. This field of study has the potential to significantly advance the development of the model. Despite the recognized constraints, this study retains a robust empirical foundation and substantive validity in its scholarly contributions.

## Conclusion

The proposed model named ZleepAnlystNet comprising feature extraction and sequential classification consists of 15 CNN models interconnected with one BiLSTM model. The 15 CNN models are categorized into three sets of CNN models named P-models, C-models, and N-models. Each set contains five class-specific models for each sleep stage: W, N1, N2, N3, and REM models, trained from specific weights of loss function for each class. Separating training was used for each individual CNN model and BiLSTM model. The overall accuracy of 87.02%, MF1 of 82.09%, Kappa of 0.8221, and the per-class F1-socres of 90.34%, 54.23%, 89.53%, 88.96%, and 87.40%, for W, N1, N2, N3, and REM respectively, are achieved. Total trainable parameters are 248,500 formulated from 211,000 of BiLSTM and 2,500 of each CNN. Approximately 2.04 s per record is spent in the feed-forwarding process. The advantages of proposed model with separating training are scalability, uncomplicate identification of significant features, facilitation of error analysis, and specific target improvements because the model can be separately analyzed. However, it comes up with substantial amounts of time spent for training multiple models.

## Methods

This study aimed to develop a deep learning model for automated sleep stage scoring based on single-channel raw EEG signal. The approach involved separating the training process into two main phases: first, the extraction of representative features, and second, the sequential learning of sleep stage classification. Additionally, the interrater reliability across three distinct scenarios was investigated comprising the scoring of the dataset conducted by experienced sleep technicians, scoring predicted by the developed model, and scoring carried out by three other sleep technicians. The developed model, named ZleepAnlystNet, consisted of three sets of CNN models, each set comprising of 5 class-specific CNN models, for representative feature extraction and one BiLSTM model for sequence classification. The input of each CNN model was the EEG signal, while that of BiLSTM model was the output of all trained CNN models including 75 features per epoch. Model performance was then evaluated and compared with the existing model using the same dataset.

### Dataset

Sleep-EDF dataset^71,72^ which are open-access public dataset was utilized in model training and evaluation processes. This dataset has been provided in 2 versions: Sleep-EDF-13 and Sleep-EDF-18. The former contributed in 2013 composed of 61 PSG recordings, while the latter contributed in 2018 composed of 197 PSG recordings. It is divided into 2 datasets separately recorded from 2 studies, which are sleep cassette or SC and sleep telemetry or ST. The SC dataset was the study of age effects on sleep in healthy participants while the ST dataset was the study of temazepam effects on sleep. Therefore, the SC dataset was selected to train and evaluate the performance of the developed model, ZleepAnlystNet. The number of files of SC dataset is dependent on the version. For Sleep-EDF-13, version 1 comprises 39 PSG recordings of 20 participants while Sleep-EDF-18 version 2 has 153 PSG recordings of 78 participants. The PSG recordings of SC dataset are composed of two bipolar EEG channels, namely Fpz-Cz and Pz-Oz with sampling rate of 100 Hz, along with one EOG channel (horizontal) with sampling rate of 100 Hz and one preprocessed submental EMG channel with sampling rate of 1 Hz. The recorded PSG files were scored according to R&K classification^10^ by well-trained sleep technicians consisting of wake, REM, stage 1, stage 2, stage 3, and stage 4, with abbreviation of W, R, 1, 2, 3, and 4 respectively. There are two stages, M and ‘?’, being movement and not scoring epochs. However, the AASM classification^11^ was implemented in this study, so the merger of stage 3 and 4 into NREM 3 or N3 was applied. M and ‘?’ stages were removed when the feature extraction model was trained prior to epoch-wise shaffling and changed to W while the sequence classification model was trained due to requirement of BiLSTM sequence learning. Only five sleep stages involving W, N1, N2, N3, and REM were included in model training and evaluation processes. The total number of epochs in each sleep stage of both Sleep-EDF-13 and Sleep-EDF-18 are shown in Table 1. In this study, all the records were trimmed 30 min before and after in-bed duration including in the model training and model evaluation because there were long periods of wake (W) at the start and the end of each record in case of 24-h recording.

The ZleepAnlystNet was designed to carry out sleep stage classification using a single-channel raw EEG signal as input. Therefore, both the Fpz-Cz and the Pz-Oz channels were separately passed through the model for model training and evaluation as the separate models.

### ZleepAnlystNet

The developed model is named ZleepAnlystNet consisting of 2 main parts which are a feature extraction part organized by a set of CNN models to pick up the representative features of each sleep stage, and a classification part that is occupied by BiLSTM model to generate sleep stages of targeted epoch.

#### CNN models for feature extraction

For representative features extraction, three sets of CNN models, named P-models, C-models, and N-models, were established to extract time-invariant features from the input data which was the single-channel EEG signal of the Sleep-EDF dataset. P-models referred to as the set of the ‘Previous’ models, while C-, and N-models referred to as the set of the ‘Current’ models, and the ‘Next’ models, respectively. Each set contains five class-specific models, named W, N1, N2, N3, and REM models (Fig. 1 on the middle column). In total, 15 distinct CNN models are involved which share the same architecture as shown in Fig. 1 (on the right column) but brought about the different weights of loss function calculating to relieve class imbalance problem. Normally, N2-stage is the dominant stage during sleep with few numbers of N1-stage. Therefore, the weights of loss function of each sleep stage were dissimilarly calculated. The designed architecture consisted of two sets of layers represented by CNN1 and CNN2. Each set of layers included a convolution layer, a rectified linear unit (ReLU) activation function layer, a batch normalization layer, and a max-pooling layer. The input data was a 1 × 3000 image which was 30-s epoch of the single-channel raw EEG and was passed through the CNN models.

The first set of layers, the CNN1, had been designed as follows: the input was firstly fed to a convolution layer composed of 10 filters with the filter size of 1 × 55, after which a ReLU activation function layer was implemented to the convoluted data. The output had undergone batch normalization to maintain stable training. Lastly, a max-pooling layer with a pool size of 1 × 16 was applied to reduce spatial dimension. The output was then forwarded to the second set of layers, the CNN2, which had been established in the same fashion as the CNN1 but different in the number of filters and filter size in the convolutional layer of 5 and 1 × 25, respectively. After the output had passed the second max-pooling layer, it was flattened and subsequently underwent two fully connected or dense layers, which were composed of neural nodes of 10 and 5 nodes, respectively, and separated by an activation function layer (ReLU). Finally, a softmax layer was applied to generate the probability of each sleep stage as a feature vector of 5 features which were prepared for sequence classification in the BiLSTM model.

All 15 CNN models had the same architecture but were trained in different data manipulations and different weights of loss function for every single class of sleep stage. Three data manipulation sets called set A, set B, and set C (Fig. 2). Set A was referred to as the ‘Current’ set. They involved the combination of the signal from the current epoch and the target which was also the sleep stage score of the current epoch. Set B was labeled as the ‘Previous’ data because it involved the combination of the signal from the previous epoch and the target which remained the sleep stage score of the current epoch. Lastly, set C was called the ‘Next’ set because the input was a signal from the subsequent epoch and the target was the sleep stage score from the current epoch. Three sets of data manipulation, set A, B, and C, occurred prior to shuffling epoch-wise to generate training and test datasets and utilized to train corresponding C-, P-, and N-models.

For each data manipulation, five class-specific models were trained, each with specific weights assigned to particular sleep stages. It can be designated as ${Net}_{c}$ where $c\in \left\{W, N1,N2,N3,REM\right\}$, i.e. ${Net}_{W},{Net}_{N1},{Net}_{N2},{Net}_{N3},{Net}_{REM}$. ${Net}_{c}$ represented the class-specific model for class $c$ which was specifically trained for class $c$ by adjusting the weight of the loss function that is particular to class $c$. The adjustment of the weight was conducted to maximize the importance of particular sleep stages from the others. The loss function used in the model training was cross-entropy loss which had been modified as in the Eq. (1):

1 $$loss= -\frac{1}{N}\sum_{n=1}^{N}\sum_{i=1}^{K}{w}_{i}{Y}_{ni}ln{\widehat{Y}}_{ni}$$

where $N$ is the total number of epochs, ${Y}_{ni}$ is the probability of epoch number $n$ that had sleep stage class $i$ which the value was either 0 or 1 due to the sleep stage score of the input, ${\widehat{Y}}_{ni}$ is the probability of epoch number $n$ that model predicted for class $i$ which was generated by softmax layer during model training process which the value was in range of 0 to 1, $K$ was 5 due to 5 classes classification, and ${w}_{i}$ is the weight used in loss function calculation for class $i$. The weighted loss function for a specific class $i$ or ${w}_{i}$ for ${Net}_{c}$ can be calculated as Eqs. (2) and (3).

2 $${p}_{i}=\left\{\begin{array}{c}\frac{{N}_{C}}{N} ;i=c\\ \frac{N-{N}_{c}}{N} ;i\ne c\end{array}\right.$$

3 $${w}_{i}=\frac{0.5}{{p}_{i}}$$

where ${p}_{i}$ is the proportion of class $i$ which $i\in \left\{W,N1,N2,N3,REM\right\}$, $N$ is the total number of epochs, and ${N}_{c}$ is the total number of epochs of the particular sleep class $c$.

#### BiLSTM model for sequence classification

After feature extraction process, which involved feeding the EEG signal into the 15 CNN models, a sequence of the output is generated. Each epoch of the EEG signal yielded 75 features produced by these CNN models. Among these features, 5 features were derived from the softmax layer of each individual CNN model (Fig. 1 on the right column at the bottom part). The sequence of 75 features per epoch from the entire EEG signal were utilized to train the BiLSTM model.

The sequence classification model was designed to consist of a BiLSTM layer with 128 hidden units to capture the temporal sequence dependencies of the input data. Following the BiLSTM layer, a fully connected layer with 5 neurons was put to pull the BiLSTM output and changed them to a lower-dimensional representation corresponding to the five sleep stages: W, N1, N2, N3, and REM. Finally, a softmax layer was placed to calculate the probability of each sleep stage, where the highest probability was determined as the predicted sleep stage. The BiLSTM architecture is shown in Fig. 1 (on the left column).

#### Training process

In this study, a separating training approach was introduced to train the proposed model. Consequently, a total of 15 CNN models and a BiLSTM model were trained separately. Each CNN model received single-channel raw EEG signal as input, sampled at a rate of 100 Hz (Fig. 2B). On the other hand, the input for the BiLSTM model comprised the outputs from the 15 CNN models, which were 75 features per epoch. These features were organized in alignment with the original sequence of epochs. For example, the first set of 75 features represented the output of the first EEG signal epoch processed by the 15 CNN models, and this pattern continued for subsequent epochs (Fig. 2C). However, certain epochs in the dataset were excluded during training of the CNN models due to their original scoring. These excluded epochs needed to be reintroduced into the sequence during training of the BiLSTM model with appropriate modifications of their label. Nonetheless, these epochs were excluded during model evaluation.

Initially, each CNN model was trained utilizing MATLAB R2022a on an Intel Core i9-10,900 CPU @ 2.80 GHz with an Nvidia GeForce RTX 2070 Super GPU. A batch size of 64 PSG-epochs was utilized for training. The validation-stop criterion was set to discontinue training when 10 consecutive validations showed no improvement. Validation occurred every 150 iterations performing one round. The maximum training-epoch was capped at 1000 training-epochs. The Adam optimizer was employed with the initial learning rate of 0.001. The data used to train the model were epoch-wisely shaffled and divided into a training set for 70% and validation set for 30% or all epochs included. Both M, and ‘?’ scored epochs were excluded during this training process.

Finally, the BiLSTM model was trained to recognize the sleep stage sequence and assign a score to each epoch. The input of the BiLSTM model was the output from feeding of EEG signal in original sequence into the 15 CNN models with respective to the data manipulation. The training process was implemented in MATLAB R2022a on the Intel Core i9-109000 CPU @ 2.80 GHz with the Nvidia GeForce RTX 2070 Super GPU. The batch size was 4 sequences which each sequence representing one record. The validation-stop criterion was triggered when 10 consecutive validations failed to show improvement. Validation occurred every 10 iterations constituting one round. The maximum training-epoch was set at 1,000 training-epochs. The Adam optimizer was applied with the initial learning rate of 0.01. The data used to train the model were divided into 90% for the training set, and 10% validation set. Both M and ‘?’ were changed to W to complete the sequence of the recording but were excluded during the model evaluation process.

### Model evaluation

To evaluate the model performance, a total of 15 CNN models and the BiLSTM model were interconnected as shown in Fig. 1 on the left column. The input signal consisted of a single-channel raw EEG signal. The signal underwent shifting process to the right resulting in the duplication of signal in the first epoch and the deletion of signal in the last epoch (Fig. 2A, set B) and was inputted to the set of the ‘Previous’ models. The original signal remained unchanged and was inputted in the set of the ‘Current’ models. Similarly, the signal underwent a leftward shift leading to the duplication of the signal in the last epoch and the deletion of signal in the first epoch (Fig. 2A, set C) and was inputted to the set of the ‘Next’ models. The model’s output was a sequence of sleep stage with respect to the original sequence of EEG signal. This classification scheme is sequence-to-sequence classification or many-to-many classification.

The assessment of model performance was evaluated employing subject-wise cross-validation, specifically the leave-one-subject-out strategy which led to the certainty that the training and test data were independent from each other. For Sleep-EDF-13, a 20-fold cross-validation was implemented which involved 19 subjects into the training set and 1 subject into the test set. This process was repeated for 20 rounds, with each subject serving as the test set once. For Sleep-EDF-18, a tenfold cross-validation was applied. During the training of the BiLSTM, both M and ‘?’ scored epochs were incorporated. However, during the evaluation of model performance, these epochs were excluded, and only the original scores of the five sleep stages were considered for assessment.

For comprehensive evaluation of the developed model feasibility, both per-class and overall parameters were utilized. Per-class metrics consisted of F1-score which was the harmonic mean of precision and recall. Precision was ratio between the true positives and all the predicted as positives and can be called positive predictive value. Recall was ratio between the true positive and relevant element or the model’s ability to correctly identify true positive, also referred to as true positive rate or sensitivity. On the other hand, overall metrics involved accuracy (ACC), which quantified the proportion of correct predictions out of the total number of the predictions; macro F1-score (MF1), the average of the per-class F1 score; Cohen’s Kappa coefficient (κ), the consistency between model prediction and sleep stage scored in the dataset; average precision or the average of per-class precision; and average recall or the average of per-class recall.

### Model analysis

The developed model was designed with a separating training approach with three data manipulations: set A, set B, and set C (Fig. 2A), to provide the time-invariant features for classification tasks. To investigate the effectiveness of the training approach and the effect of data manipulation for feature extraction, two separated scenarios were consequently used as mock-ups including scenario A and scenario B, which referred to as separating training and end-to-end training, and several combinations of sets of data manipulation, respectively.

#### Efficacy of separating training and end-to-end training

For scenario A, the effectiveness of the training approach was investigated in benefits between the training approaches. In case of separating training, a single CNN model which the architecture was the same as represented in Fig. 1 (on the right column) was trained separately from BiLSTM. Consequently, the BiLSTM model with the same architecture represented in Fig. 1 (on the left column) was trained. Only data manipulation set A was utilized in this scenario. The hyperparameters of both CNN and BiLSTM models used during training process were the same as reported in the ‘Training process’ sub-section. However, the weighted loss function due to sleep stage was not applied in this case. Therefore, five features in total were used as the representative features to train BiLSTM model. On the other hand, in case of end-to-end training, both CNN and BiLSTM models were interconnected, maintaining the same architectures as in the case of separating training. The hyperparameters and the training process were the same as CNN training explained in the ‘Training process’ sub-section. The Fpz-Cz channel of Sleep-EDF-13 was the input of this mock-up simulation.

#### Efficacy of sets of data manipulation in CNN training

For scenario B, the effectiveness of data manipulation used for the training of CNN models was assessed. To remind, the developed model consisted of 15 distinct CNN models which were allocated by 2 factors. The first factor was class-specific in CNN models which the models were trained by adjusting the weighted loss function and they were divided into 5 sets. The second factor was data manipulation divided into 3 sets (Fig. 2A). For instance, our proposed model consisted of 15 CNN models and BiLSTM model connected. Of which 15 CNN models were three sets of data-manipulated models called P-models, C-models, and N-models which abbreviated from the set of the ‘Previous’ models, the set of the ‘Current’ model, and the set of the ‘Next’ models, respectively. Each set of data-manipulated models contained five class-specific models which were trained with the weighted loss function adjusting to the corresponding sleep stage classes. Each data manipulation set was used to train their respective set of data-manipulated models. The details were provided in the ‘CNN models for feature extraction’ sub-section.

Testing conditions were established based on seven sets: using only set A, set B, and set C; using combinations like set A + set B, set A + set C, set B + set C, and set A + set B + set C. Each condition used a particular set of data to train the corresponding models. For example, in the condition set A + set B, data set A was used to train the set of the ‘Current’ models, and data set B was used to train the set of the ‘Previous’ models. All three sets of the ‘Previous’, ‘Current’, and ‘Next’ models were composed of five class-specific models with the weighted loss functions. All conditions employed separating training approach. The training process was explained in the ‘Training process’ sub-section. The performance was considered using the Fpz-Cz channel of Sleep-EDF-13.

### Manual scoring by sleep technician

All PSG files in Sleep-EDF-13 were separately scored by three well-trained sleep technicians. A total of 39 files in the SC study were scored once again with blinding of the sleep stage scoring from the dataset by every sleep technician according to the AASM scoring manual^11^.

### Cross-dataset validation

Another dataset called the Sleep Heart Health Study (SHHS) was utilized for cross-dataset validation. The SHHS was a multi-center cohort study implemented by the National Heart Lung and Blood Institute to determine the cardiovascular and other consequences of sleep-disordered breathing^73,74^. This dataset was composed of 2 subsets which were SHHS1 and SHHS2 consisting of 6,441 and 3,295 recordings respectively. The former, SHHS1, was contributed between 1995 and 1998. The latter, SHHS2, took place between 2001 and 2003. The PSG recording was composed of 2 EEG channels which were C3-A2 and C4-A1 with sampling rate of 125 Hz; right and left EOGs at sampling rate of 50 Hz; a submental EMG sampled at 125 Hz; and other standard PSG signals. The scoring procedure of this dataset was conducted under the R&K standard^10^, therefore the same combination procedure of N3 and N4 was applied to take totally 5 sleep stages in the classification task. The dataset used in the cross-dataset validation process was evenly random from both SHHS1 and SHHS2 for 200 recordings in total having 100 recordings each.

All trained models by the Fpz-Cz and the Pz-Oz channels of both the Sleep-EDF-13 and the Sleep-EDF-18 datasets. The Sleep-EDF-13 dataset contributed 40 models, with 20 models for each channel, while the Sleep-EDF-18 dataset contributed 20 models, with 10 models for each channel. These models were employed for sleep stage classification of the randomized 200 records from the SHHS dataset to simulate an unseen data situation. Model evaluation used both C3-A2 and C4-A1 as input channels. Overall and per-class evaluation parameters were consequently used to investigate the model performance. Following evaluation across all folds, the fold yielding the highest performance in each condition was selected, resulting in four models, two from the Sleep-EDF-13 dataset and two from the Sleep-EDF-18 dataset. Subsequently, all 6,441 records of the SHHS1 dataset were fed into these four models to evaluate performance. This cross-dataset validation process aimed to investigate the feasibility of generalization of the separating training approach which is the focus in this study. Therefore, a fully systematic evaluation was not included in this study.

Moreover, the developed model underwent additional training using the randomized 200 records from the SHHS dataset, with both C3-A2 and C4-A1 employed as input channels for both model training and evaluation. This process resulted in the creation of two new models. Subsequently, a five-fold cross-validation process was employed to evaluate the performance of these newly trained models. The total number of epochs of all 200 recordings from SHHS dataset is exhibited in Supplementary Fig. S1C.

### Statistical analysis

Descriptive statistics were utilized to express the model performance explained in ‘Model evaluation’ and ‘Cross-dataset validation’ sub-sections which are comparable to the existing sleep stage scoring models in the literature. Cohen’s kappa statistic was used to interpret the interrater reliability between scoring in the dataset and the model prediction. Fleiss kappa statistic was used to interpret the interrater reliability of more than 2-rater conditions which consisted of (a) among 3 sleep technicians, (b) between scoring in the dataset and 3 sleep technicians, and (c) between the model prediction and 3 sleep techniques. Kappa coefficient was calculated with 95% confidence intervals. The degree of agreement was considered as None (κ = 0 − 0.20), Minimal (κ = 0.21 − 0.39), Weak (κ = 0.40 − 0.59), Moderate (κ = 0.60 − 0.79), Strong (κ = 0.80 − 0.90), and Almost Perfect (κ was above 0.90) according to McHugh^67^. A two-tailed z-test was conducted to evaluate the coincidence of reliability. A *p*-value of less than 0.05 was considered significant.

### Supplementary Information

Supplementary Information 1.

Supplementary Information 2.

Supplementary Information 3.

Supplementary Information 4.

Supplementary Information 5.

Supplementary Information 6.

Supplementary Information 7.

Supplementary Information 8.

Supplementary Information 9.

Supplementary Information 10.

Supplementary Information 11.

Supplementary Information 12.

## Data Availability

Sleep-EDF-13 and Sleep-EDF-18 datasets have been archived with appropriate blind identification process. Access for the datasets were obtained via online: https://www.physionet.org/content/sleep-edfx/1.0.0/. SHHS have been archived by the National Sleep Research Resource. Access for download in this URL: https://www.sleepdata.org/datasets/shhs.

## Code availability

The code used for model training was generated with Deep Network Designer app in MATLAB. The detail of model architecture in the Methods section was sufficient to be independently replicated. The model evaluation process was a simple code to generate confusion matrix from the testing dataset. Therefore, the code was not provided due to sufficient detail for replication.
