# Supplementary figures and images for: ZleepAnlystNet: a novel deep learning model for automatic sleep stage scoring based on single-channel raw EEG data using separating training
Source: Sci Rep. 2024 Apr 29;14:9859. doi: 10.1038/s41598-024-60796-y (PMC11058251; doi:10.1038/s41598-024-60796-y)

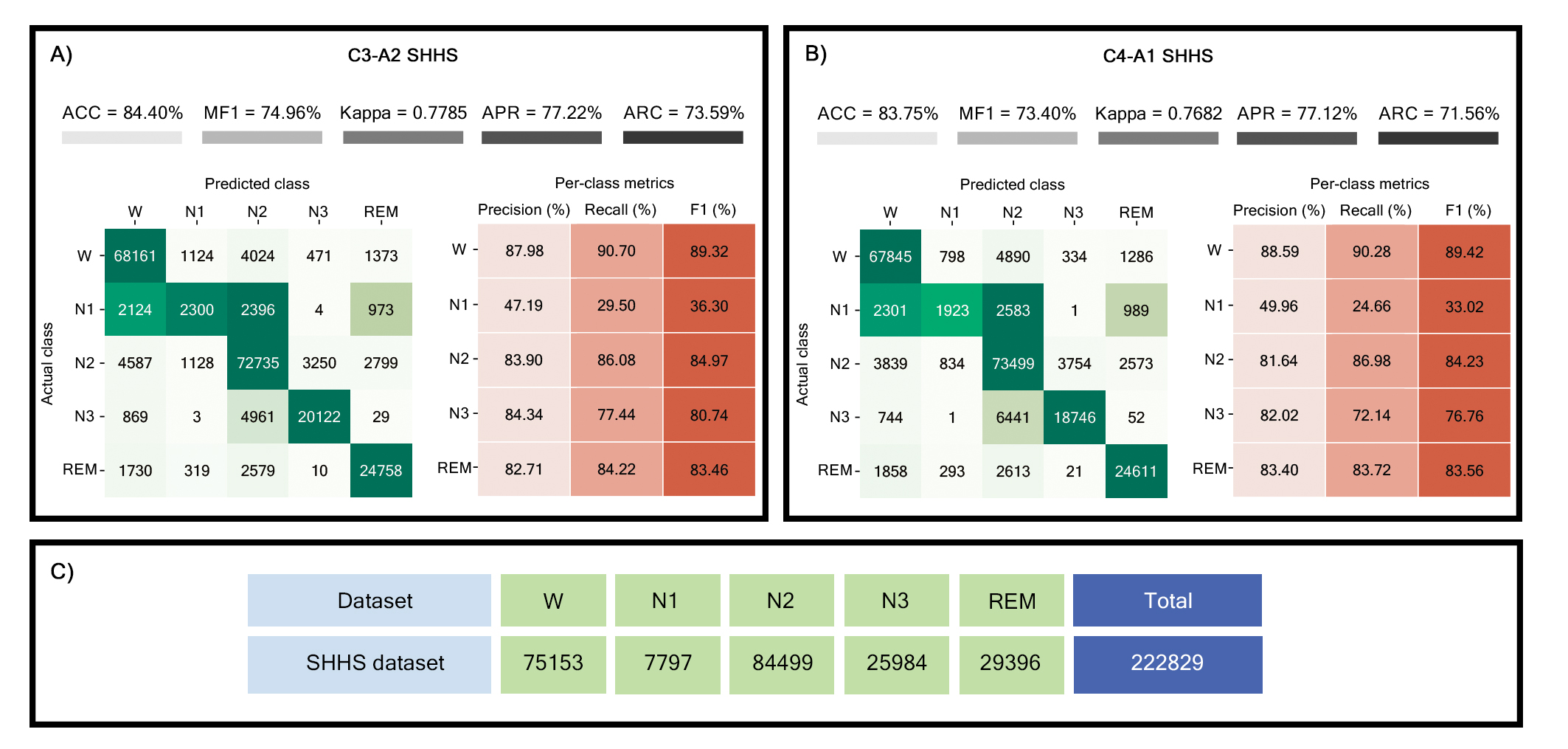

Supplement: Supplementary file 1 — Supplementary Information 1. [file 41598_2024_60796_MOESM1_ESM.jpg]

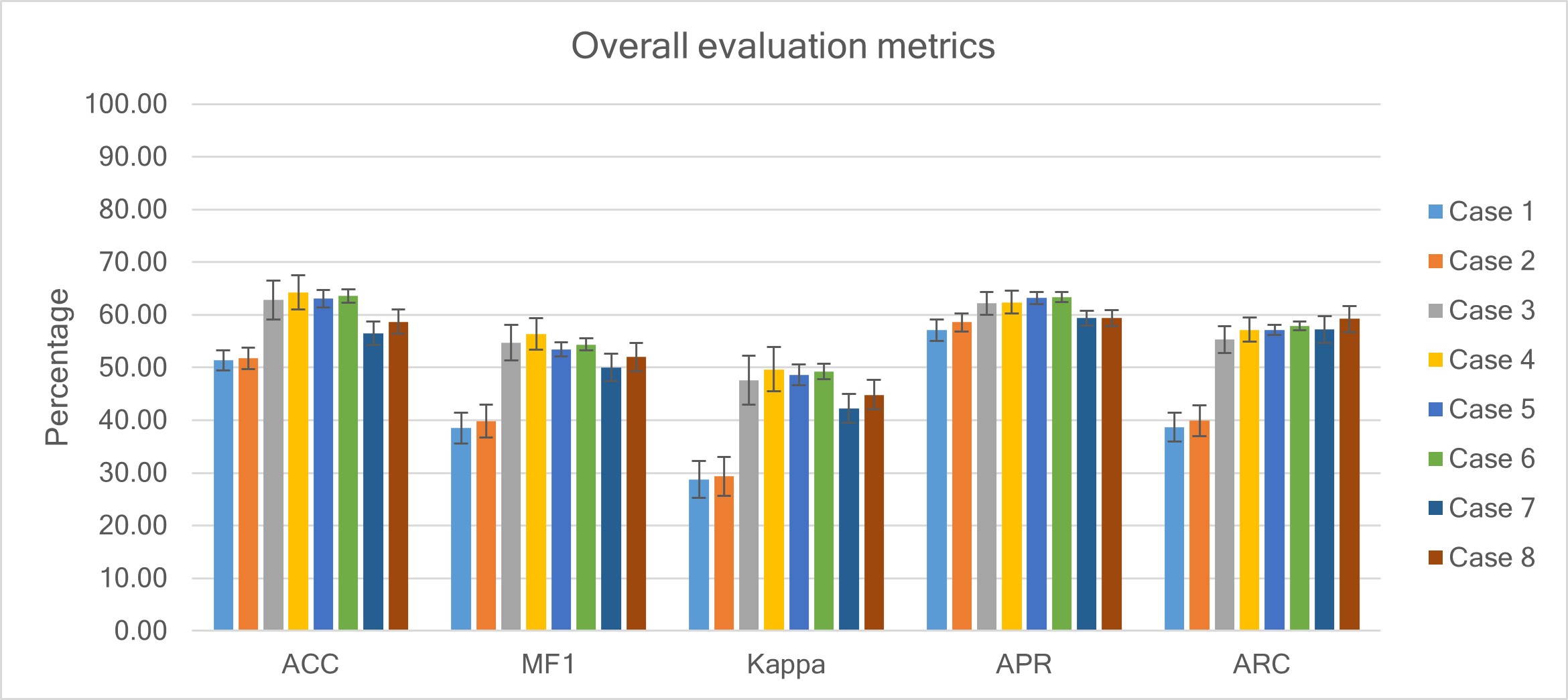

Supplement: Supplementary file 2 — Supplementary Information 2. [file 41598_2024_60796_MOESM2_ESM.jpg]

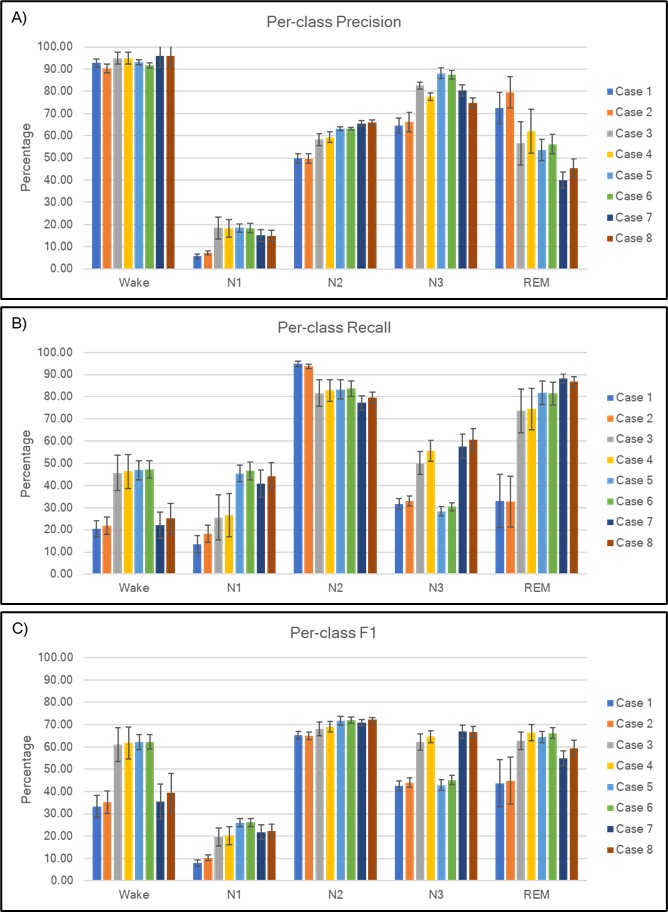

Supplement: Supplementary file 3 — Supplementary Information 3. [file 41598_2024_60796_MOESM3_ESM.jpg]
