# Supplementary material for: ZleepAnlystNet: a novel deep learning model for automatic sleep stage scoring based on single-channel raw EEG data using separating training
Source: Sci Rep. 2024 Apr 29;14:9859. doi: 10.1038/s41598-024-60796-y (PMC11058251; doi:10.1038/s41598-024-60796-y)
Supplement: Supplementary file 12 — Supplementary Information 12. [file 41598_2024_60796_MOESM12_ESM.docx]

**List of supplementary figures**

**Supplementary figures legend**

Supplementary figure 1: Model Performance. The confusion matrix, overall evaluation metrics, and per-class evaluation metrics of the developed model trained using 200 recordings from SHHS dataset are presented. A) Depicts results obtained from the C3-A2 channel of the SHHS dataset. B) Shows results obtained from the C4-A1 channel of the SHHS dataset. C) Illustrates the dataset used and the number of epochs utilized in model training and evaluation.

Supplementary figure 2: Average Overall Evaluation Metrics with Standard Deviation. This figure displays the average value of overall evaluation metrics along with the standard deviation for each combination between train and test datasets in cross-dataset validation. Different cases indicate pairing between training data and evaluating data, representing as training & evaluating. Case 1: Fpz-Cz Sleep-EDF-13 & C3-A2 SHHS; Case 2: Fpz-Cz Sleep-EDF-13 & C4-A1 SHHS; Case 3: Pz-Oz Sleep-EDF-13 & C3-A2 SHHS; Case 4: Pz-Oz Sleep-EDF-13 & C4-A1 SHHS; Case 5: Fpz-Cz Sleep-EDF-18 & C3-A2 SHHS; Case 6: Fpz-Cz Sleep-EDF-18 & C4-A1 SHHS; Case 7: Pz-Oz Sleep-EDF-18 & C3-A2 SHHS; and Case 8: Pz-Oz Sleep-EDF-18 & C4-A1 SHHS.

Supplementary figure 3: Average Per-Class Evaluation Metrics with Standard Deviation. This figure presents the average value of per-class evaluation metrics along with the standard deviation for each combination between train and test dataset in cross-dataset validation. A) Illustrates per-class precision. B) Depicts per-class recall. C) Shows per-class F1-score. Different cases indicate pairing between training data and evaluating data, representing as training & evaluating. Case 1: Fpz-Cz Sleep-EDF-13 & C3-A2 SHHS; Case 2: Fpz-Cz Sleep-EDF-13 & C4-A1 SHHS; Case 3: Pz-Oz Sleep-EDF-13 & C3-A2 SHHS; Case 4: Pz-Oz Sleep-EDF-13 & C4-A1 SHHS; Case 5: Fpz-Cz Sleep-EDF-18 & C3-A2 SHHS; Case 6: Fpz-Cz Sleep-EDF-18 & C4-A1 SHHS; Case 7: Pz-Oz Sleep-EDF-18 & C3-A2 SHHS; and Case 8: Pz-Oz Sleep-EDF-18 & C4-A1 SHHS.
